# Book Reviews

**Published:** 1818-03

**Authors:** 


### CRITICAL ANALYSIS

### *OF RECENT PUBLICATIONS,*

IN THE

### DIFFERENT BRANCHES OF PHYSIC, SURGERY, AND MEDICAL PHILOSOPHY.

---

*Medico-Chirurgical Transactions, published by the Medical and Chirurgical Society of London.* Vol. VIII. Part II.—Longman and Co. 1817.

*On the Pellagra, a Disease prevailing in Lombardy.* By HENRY HOLLAND, M.D. F.R.S. (See our Collectanea.)

*Observations on the Treatment of Syphilis, with an Account of several Cases of that Disease, in which a Cure was effected without the use of Mercury.* By THOMAS ROSE, Esq. A.M. of Baliol College, Oxford, Surgeon to the St. James's Infirmary, and to the Coldstream Regiment of Guards.

*Observations on the Treatment of the Venereal Disease without Mercury.* By J. G. GUTHRIE, Esq. Deputy Inspector of Military Hospitals, Surgeon to the Royal Westminster Infirmary for Diseases of the Eye, Lecturer on Surgery, &c.

OUR Collectanea of the present month will be considered as subsidiary to the above, and also to a succeeding article in the Edinburgh Journal. It may be said, that all we have produced, and much more, may be found in every library, and has been repeatedly brought before the medical reader. But, medical men either do not read, or they read in too great a hurry to digest. We will not say how far Mr. Hunter's example may be pleaded,—by some he is said not to have read at all, and by others to have read more than he confessed. We consider neither of these allegations well-founded: whoever peruses what he has left in print, will find that he never passes over a writer of any reputation on the subject of his inquiries. He had, besides, the largest opportunity of knowing not only all that was published, but all that was discovered and unpublished throughout Europe. His brother had the finest medical library then known in London, was a scholar and a reader, and surrounded by scholars and readers. Mr. Hunter's house was the focus of all physiological intelligence; so that, even during his misunderstanding with, and after the demise of, William, John had every means of learning what was written by the dead or discovered by the living; and it is plain that he availed himself of all.

Before we proceed any further, we shall transcribe the introductory part of Mr. Rose's paper, which may serve us as a text on which the reader must permit us to make an ample commentary.

"The existence of a numerous class of diseases, whose symptoms bear a close resemblance to those arising from the venereal poison, but which are curable without the exhibition of mercury, has for many years been satisfactorily ascertained, and is confirmed by the repeated experience of every surgeon. To form an accurate diagnosis between these and syphilis, is in many cases extremely perplexing; and the attention which has of late been bestowed on this important subject has only shewn more clearly the difficulties in which it is involved.

"It unfortunately happens that, unlike the effects of other morbific poisons, the symptoms of syphilis are not so marked and peculiar in their character, that by them alone the presence of its virus can be ascertained. Precisely similar ones occur in other diseases, and are even, in certain habits, induced by the action of mercury; and it is only in their history and progress that any difference is to be detected. These are frequently so confused by the inaccuracy of patients and other causes, that no reliance can be placed on them, and we shall find, in practice, that there are few cases in which the nature of the disease is sufficiently decided not to admit of some degree of doubt being entertained on the subject. Under such circumstances, we cannot wonder at the confused accounts which we meet with of this disease, and at the vague and discordant opinions which have at all times prevailed respecting it; nor, whilst its diagnosis rests on such obscure and unsatisfactory grounds, can we expect to find any consistent rules established for its treatment Two points, however, appear to be generally agreed upon: namely, that syphilis does not admit of a natural cure; and that mercury is the only remedy hitherto known which has the power of destroying its virus. So fully are these supposed to be established, that, where a disease has been cured without the use of that medicine, and has not afterwards returned, such fact alone, whatever may have been the symptoms, is regarded as sufficient proof that it was not a case of syphilis.

"That mercury, where its action is properly regulated and kept up for a sufficient time in the system, effectually destroys the venereal virus, is confirmed by the fullest experience. Its utility, both in the local and constitutional forms of the disease, is too obvious to admit of a question; but the absolute necessity for its employment is more difficult of proof, and seems to have been assumed on less satisfactory evidence. It is true, the majority of practitioners have concurred in this opinion during a long series of years: it may be said to be founded on the experience of more than three centuries. But this, although a strong argument in its favour, will not justify our yielding an implicit assent to a proposition so general and so important. The notions formerly entertained of this disease are known to have been extremely incorrect; and it should not be forgotten that the same argument is not less conclusive as to the necessity of a course of mercury in multitudes of cases, which experience has now fully shewn to be even aggravated by its use."

We have often regretted the misfortune it proves to the writer, as well as his readers, when the former has the gift of an easy and fluent style. In whatever he undertakes, he meets with few difficulties, and the reader is relieved from the dryness of close reasoning. Every thing is perspicuous where nothing but the exterior is shewn; and every thing pleasing where the exhibition is not only in the most glowing, but in the most agreeable, colours. *Et prodesse et delectare* is always within the power of the painter and the poet, to whom *quidlibet audendi semper fuit æqua potestas.* We will admit there are certain rules which they must not transgress; but still the external and the more obvious appearances are all that are required of them, and they are left to the choice of their subjects, and even to the manner in which they chuse to represent them. The philosopher has a different task: he must prove every thing as he goes on, and this is often in his power even to a demonstration. The motions of the heavenly bodies are ascertained to a mathematical exactness, and this can only be the work of a few, who must be judged by competent peers; the discriminating marks of plants and animals are with more facility ascertained, and are for the most part permanent: but such is the variety in disease, and so transient are its appearances, that the same complaint, even in the same subject, is not always exactly similar for two succeeding days. Morbid poisons, it is true, have a more decided character than most other local complaints. Those which are chronic are more permanent in their figure, yet even these will vary from a peculiarity of constitution, and still more from the application of remedies. To trace all these varieties, and even to ascertain the limits which separate the local actions excited by different morbid poisons, is a work which could only be accomplished by one whose genius seems directed to the true method; and even to follow the directions we receive from him requires, at least, a temporary surrender of our judgment, with the application of all our mental energies to this single object. In this light we view a Hunter.

In our remarks on the passage extracted from Mr. Rose's paper, we shall admit that the attention which has *lately*

been bestowed on this subject has added to the difficulty of diagnosis, but we cannot allow that it has shown it "more clearly;" nor can we allow that the symptoms of syphilis are not sufficiently peculiar in their character. Here we must refer to our common practice of demanding the meaning of words: first, what is meant by "morbific poisons;" next, by syphilis. Is it not the very distinguishing character of *every poison* to induce disease? and where is the difference between morbific and that which induces or *makes a disease*? We presume the word is intended as synonymous to, or an improvement of, Mr. Hunter's term—*morbid poison*. But the two expressions have an entirely different meaning. Morbid is used in distinction to that which is in health. Some animals are endued with poisons during their own *sound health*; whereas, a *morbid* poison is only known by a *diseased* action in the animal that inflicts, as well as in the animal that receives it. The word syphilis was formerly confined to what was called the second infection,—to what Mr. Hunter called the secondary symptoms, and to those he confined the term. This would be of less consequence, were it not for the position connected with it,—namely, that its character is not well marked. The secondary symptoms are certainly often distinguished with difficulty from those of other morbid poisons; but the primary ulcer, or chancre, has a more perfect character than any other morbid poison, excepting the cow-pox.

Next, we must ask, what is meant by the expression—"that mercury, where its action is properly regulated, and [that action] kept up for a sufficient time in the system, effectually destroys the venereal virus?" We would ask, who can regulate the action of mercury? We are aware the meaning of the author is—"the action excited in the system by mercury;" and that, instead of destroying the virus, his meaning is—that it supersedes permanently, or destroys, the *action excited in the system by* the virus. These distinctions may appear trifling to some of our readers, but not to those who take the trouble of studying the laws of those actions which are excited in the system by different stimuli: for we scruple not to assert, from a practical confirmation of Mr. Hunter's doctrine, that if, by any course which permanently cures a chancre, we expect to prevent the appearance of secondary symptoms, we may find ourselves mistaken. We mention this more particularly, because Mr. Rose seems to suspect that, in the opinion of some, there may be more danger of secondary symptoms where the primary are cured without mercury. For our parts, satisfied as we are that the primary symptoms from all morbid poisons are more severe than the secondary, we should not give ourselves any concern about the latter if the first had been cured without mercury.

Last of all, we must give our decided negative to the suggestion, that "the distinction between syphilis and syphilöid (the reader will pardon our transcribing the latter term,) diseases has been made to depend so much on the former admitting of no cure excepting by mercury." We admit, where the character has been generally similar, such a proof may be admitted as presumptive. But that there are other diseases known to arise from morbid poisons, and curable only by mercury, has been long since established; nor are we by any means certain, that even the secondary symptoms of the venereal disease, when confined to the skin, may not be cured without mercury. This is at least probable, from the very slight mercurial irritation to which they yield in their early stage. The trial is not indeed easily made, nor is it worth an experiment. In an advanced stage, it is probable that there is no constitutional irritation we could venture to excite, excepting by mercury, which would be sufficient; but in this respect syphilis remains only on the same ground as the sivvens or sibbens of Scotland.

Mr. Rose proceeds,—

"Other specifics have been occasionally tried in the cure of this disease, at every period since it is considered as having made its first appearance, and the success of some of them has been attested, after ample experience, by men of veracity and of acknowledged abilities. It is highly probable that many of the cases supposed to establish the efficacy of these remedies were not venereal; and, from their uniformly having fallen into disuse, we may take it for granted, that the remedies themselves were not possessed of all the virtues attributed to them; but, if no instance of syphilis can be cured without a course of mercury, can we possibly account for a single *rational* practitioner having been deceived by them?"

We perfectly agree, that no *rational* practitioner would be deceived, if by such a practitioner is meant one who *reasons* with an attentive consideration of all the evidence. If, then, these remedies have fallen into disuse by those of whom we have the highest opinion, and who have had the most extensive opportunities of judging, we may fairly assert, that the most rational and the best informed practitioners have seen the necessity not only of giving mercury, but even of exciting a very high irritation for the cure of a primary ulcer, at all inveterate. What is the whole of Mr. Pearson's book but a confirmation of this important fact? It is true, Mr. Pearson enters into no close reasoning, and that his descriptions are by no means minute. But, however, we may consider his book as a mere relation of facts, which is all the author undertakes; such is not the case with Mr. Hunter. In him we meet with close descriptions, with reasoning so truly philosophical, that, to be understood, he must be studied. It is well known to his contemporaries, that Mr. Hunter was more backward in administering mercury in all doubtful cases than any surgeon of his days; that he never willingly prescribed it till he found the true character of the chancre. Not that he was ignorant of the virtues of that mineral in many other local diseases, whether arising from morbid poisons or not; but because, like a true philosopher, he was anxious to establish certain laws, which he saw could only be done by watching the progress of an ulcer without altering its character by so powerful a remedy. It is the contrary method that has produced all the confusion of the present day. Since a practice has been introduced of giving *alterative* doses of mercury for all diseases, local or constitutional, every ulcer on the genitals has been treated in this manner, and, if not found to yield to the smaller doses, a higher irritation has been excited, and the disease has been considered venereal. We shall not stop to notice the authority on which this practice is founded and supported. It is enough for us to remark, that it is purely experimental, without leading to any philosophical certainty. Can we wonder then if it has led to the present confused state of things, of which we may truly say,

"Nec quot confundis vel JURA aut NOMINA sentis."

"In several parts of Europe, (continues Mr. Rose,) mercury is either not used at all, or administered in such a manner as we should consider totally inadequate to the cure of the disease.

"I shall select only a single instance: in the Fourth Volume of the Transactions of this Society, a curious account will be found by Mr. Fergusson, of the treatment of the venereal disease in Portugal. It appears, that the use of mercury in that complaint is there almost entirely abandoned, and the consequences have not been such, as, according to our ideas, might naturally have been expected. It is reasonable to think, that a considerable part of the cases of which Mr. Fergusson speaks, would not be regarded by cautious practitioners as venereal. In mentioning the effects of the disease on the British soldiers, he states, that 'the constitution, while strongly under the influence of mercury, became affected with the secondary symptoms in a proportion that could not have been expected,' and some other circumstances which would lead us to believe, that several of the cases must at least have been of a doubtful nature; but the fact itself, that mercury is hardly at all employed in that country for the cure of any such diseases, is certain, not only from Mr. Fergusson's account, but from the observations of the numerous British surgeons who have served there; and, when we reflect on the well-known contagious properties of the syphilitic virus, we shall find it difficult to comprehend, why, when thus left to itself, it should not be productive of far more serious mischief.* If we compare the account of this disease in Portugal, with what Mr. Hunter considered the natural result of its introduction, where the means of cure were not understood, or with the description of its ravages when it is imagined to have first shewn itself at the close of the 15th century, the contrast cannot fail to strike us. In endeavouring to prove that it was taken to the Island of Otaheite, by some of the crew of M. Bougainville, and not by that of Captain Wallis, Mr. Hunter remarks, that, 'as M. Bougainville arrived at the island ten months after Wallis, there was a sufficient time, if any one of the crew of the latter had been diseased, for the inhabitants of the whole island to have been infected; and the ravages of the disease must have been evident to the crew of Bougainville immediately upon their arrival.' The ac-

---

* "I had several opportunities in the course of the Peninsular war, of witnessing the little attention paid to this disease in the earlier stages, both by the Portuguese and Spaniards. I was able to trace some of the natives of those countries (who were attached to the commissariat,) in perfect health for two or three years, after sores, which I had supposed to be venereal, had been healed without mercury. The use of that remedy had been prevented both by their own unwillingness to have recourse to it, and by the almost constant exposure to the open air, both during the night and day, which the care of their mules required of them. A few similar instances came under my observation amongst our own soldiers, where the use of mercury was interrupted at early periods by movements of the army or other causes, and was not afterwards resumed. I have often wondered, that, not in one of these, any ill effects ensued; but I could only infer, that my opinion of the nature of the disease had been erroneous, although, in the cases to which I allude, it had by no means been hastily formed, and the sores had had every character of true chancres.

"A few instances of nodes, and caries of different bones, are to be met with in Portugal, as in every part of the world; but they did not appear to me at all more frequent there, than in any other country I have had an opportunity of visiting. I examined some of those objects, but did not chance to meet with a single one, where the history of the disease corresponded with the idea of its being syphilis. It may be remarked, that, although mercury is scarcely thought of in that country for the cure of primary sores, or of such ca es where its vir ues are undoubted, it is often had recourse to, very unmercifully, in those diseases in which its effects are most pernicious.

counts of the destruction occasioned by the venereal disease in Otaheite, appear by subsequent observations to be entirely unfounded; but they are referred to here, because, if the commonly received ideas on the subject are correct, such would certainly be the consequences of that poison being introduced into any country, where the sole antidote to it was either unknown or disregarded; whilst the well authenticated description of its mild effects in Portugal, when not interrupted by mercury, is totally irreconcileable with these ideas. Mr. Carmichael supposes, from the description given by Mr. Fergusson, that the disease which prevails in Portugal, is a phagedenic or sloughing ulcer, differing from the true syphilitic chancre, and in which he has not found mercury to be either necessary or serviceable; but in what manner that kingdom, over-run by every description of foreigners, has escaped the introduction of syphilis itself, is left entirely to conjecture.

"3. Mr. Fergusson mentions in the same paper a fact, which I had also heard from others, that, in the German regiments in our service, some surgeons, 'pertinaciously, even officially refused to prescribe mercury in syphilis, asserting that it was not necessary to the cure.' 'Such alarming conduct,' he adds, 'as being referable only to the most brutal ignorance, of course met with no quarter.' A similar circumstance was communicated to me by the late Dr. Banks, who had served some time in the Mediterranean. He assured me, from his own observation, that the surgeon of one of our foreign regiments* to which he himself was attached, used no mercury for several years in venereal complaints, and believed that secondary symptoms did not occur except where that medicine was employed."

When rational practitioners confound diseases in such a manner; when one high in the service expresses his astonishment at the brutal ignorance of those who cure diseases by the safest and most simple remedies, it is not for us to set them to rights. We feel, however, some relief, from the mention of Mr. Hunter. At the sight of his name we fancy ourselves again within the beacon of sound reason and accurate description. Whoever peruses his remarks on the diseases at Otaheite will at once perceive his caution and his candour: he could hardly believe that every account, whether from medical men or others, was erroneous; but it is easy to perceive the difficulties he was under in reconciling the supposed facts with his doctrines. Subsequent observations from one on the spot, and well acquainted with Mr. Hunter's doctrines, proved that the disease had never existed

* "I believe he said the Maltese regiment; but I forget exactly which he named. Dr. Theod. Gordon perfectly recollects the conversation to which I allude."

in that island. What is more to the purpose, we find, that one who had never visited the place, but had received more recent and more minute descriptions of the diseases which prevail in the South Sea, was satisfied that the venereal, as described by Mr. Hunter, was not among them.* After this, shall we say, that " there is nothing so characteristic in a chancre as to furnish incontrovertible proof of its nature." It is true that sores are afterwards described by Mr. Rose, which seem to have the character of the true chancre, and which, besides the author's, have the authority of others as respectable, and better known by their longer residence in the metropolis. To this we shall only answer, (though we might not have ventured to offer our own judgment, unsupported by rules which are to be met with in Mr. Hunter,) that we have not found those difficulties in detecting the true venereal chancres, and that the manner in which they have yielded to mercury has confirmed our opinion.

Many of the cases which follow (twenty-eight in number) confirm a doctrine of Mr. Hunter, which few seem to recollect, namely, that new morbid poisons are perpetually arising, many of which never being communicated any further, cease with the first person who is infected. Many of the sores described, were with hard edge and basis. We know the uncertainty of description, and how minutely and frequently every ulcer must be examined, in order to ascertain its true character. An ulcer on a part exposed to friction will often assume such a surface in its own defence.† It is not, when we see an ulcer on a part seldom quite at rest, that we can judge of its character; yet, such was the condition of the ulcers here described. Many others had no claim to the venereal character; most of them, in their early stage, were attended with fever; and many, after sloughing, healed immediately. This peculiarity of attendant fever on some of these prisores, and also skinning immediately after sloughing, has been considered by several writers as sufficient to distinguish them from the true chancre.

" In one case (says Mr. Rose) of phagedenic ulcers, the whole of the glans penis was destroyed, and, in another, a considerable portion of it. These cases I shall describe, as they were the only ones in which any degree of permanent deformity was produced. In both, there was such disturbance of the system from the moment of

---

* See Edinburgh Medical and Surgical Journal, Vol. II. and Vol. VIII. pages 157 and 419.

† See Mr. Hunter's ingenious Doctrine on the Cause of this Peculiarity in the True Chancre, p. 320, 8vo. edition.

their admission, that the use of mercury would have been highly improper. I have several times seen that medicine exhibited under such circumstances,* and it has always appeared to me to hurry on the ravages it was intended to check. The same remark applies to the early use of bark, wine, and any tonics or stimulants. I am not at all aware whether such sores are produced by the syphilitic poison or not. They are seldom followed by secondary symptoms, but this has been accounted for, by the parts contaminated being so rapidly destroyed. They appear at any rate to be occasioned by the application of some morbific matter, and it is not easy to explain, whether the greater degree of erethismus excited by the local affection, should be attributed to any peculiarity in that matter, or is owing to the peculiar state of constitution of the person infected. Mr. Fergusson gives a case of this nature, in which he says, 'the infection was communicated by an opera dancer at Lisbon, apparently in perfect health, who continued on the stage for several months afterwards, occasionally infecting others, without any thing extraordinary, as far as he could learn, in the nature of the symptoms.†"

Whoever the surgeon was that administered mercury in such a disease, ought, by way of penance, to read Mr. Hunter till he can repeat his catechism; and even then, we fear, he would, like most school-boys, repeat without understanding. Several cases follow, in which slough with fever was the only symptom. Whoever should treat such diseases with mercury after reading Mr. Carmichael, deserves a still severer penance. There may, however, be some allowance for a practitioner's ignorance of this last-named respectable author; but whoever neglects to make himself informed of the writings of one not only universally esteemed, but quoted by the faculty; one who first reduced this disease to certain laws, and pointed out the anomaly before confounded with it;—whoever, we affirm, practises without all the information he can derive from such a source, seems to us like a mechanic without a knowledge of mathematics, or a lawyer without Coke upon Littleton. Each, by what he picks up in the routine of practice, may acquit himself tolerably well on common occasions, but neither can be prepared for any thing further.

Though we have mentioned only primary symptoms, yet many of the cases described by Mr. Rose, were followed by

---

"* I have at present a patient under my care in the St. James's Infirmary, who had used mercury for eight days in such a description of sores. The whole glans was destroyed, and the disease was making rapid progress under that treatment. The greatest benefit was experienced from bleeding, and a change of system."

"† Medico-Chirurgical Transactions, Vol. IV. p. 12."

ulcers in the throat, and various affections of the skin, viz. papulæ, lichens, vesicles, erythema, psoriasis, nor was iritis wanting, sometimes attended with inflammation in the cornea or conjunctiva. There is often much difficulty in distinguishing a venereal sore throat. As to the appearances on the skin, we can only suppose that they were imputed to a morbid poison by their commencing with copper-coloured spots. If they afterwards assumed these various forms, we should impute each to a different morbid poison. We confess ourselves unable to make this distinction concerning the syphilitic iritis, nor can we find any rules in Mr. Saunders' to direct us. He admits, indeed, such a case; but it is on the presumptive evidence of other symptoms. Mr. Rose adds, in a note—"This important fact has been clearly explained in Dr. Farr's last edition." Yet, in the text, we read—"but the diagnosis is not satisfactory." However, mercury is said to be useful in both cases. We still, therefore, remain in doubt, whether the eye is ever affected by that poison.*

Having stated his cases, with short remarks on each, Mr. Rose concludes with some observations, the most important of which we shall extract.

"The impossibility of effecting a cure of syphilis, except by the exhibition of mercury, has, for so long a period, been admitted as an established principle, and has been so generally adopted as a leading characteristic of the disease, that I cannot expect the facts and cases which I have related, to do more than lead to a further investigation of so intricate and important a question. If that principle is erroneous, new views of the subject, of material consequence, will evidently be opened to us; and part of the difficulties which have been met with in distinguishing between syphilis and some other diseases, will naturally be accounted for, by the mistaken grounds on which a distinction has often been looked for, where none in reality existed.

"That the diseases which are commonly communicated by sexual intercourse, do not all arise from one peculiar poison, is an opinion which can hardly be doubted. Long before syphilis appeared, some of them were frequently met with, and Mr. Pearson thinks, that, in addition to those formerly known, new forms of disease have occasionally arisen, 'which are succeeded by a regular series of symptoms nearly resembling the progress of lues venerea.†' How far the variety which we meet with, in the symptoms of venereal cases, is to be attributed to different poisons; or how far the symptoms of the same poison may be modified, and al-

---

* See Hunter, p. 483, 8vo.

"† Vide Observations on the Effects of various Articles of the Materia Medica in the Cure of Lues Venerea. Second Edition, Introduction, p. 53."

tered by constitution, climate, and habits of life, is as yet merely hypothesis. We have seldom an opportunity of tracing different cases to the same source of infection, and of comparing their progress with each other, under such circumstances, to see how nearly they would correspond. Inoculation, if admissible, would throw much light on this interesting question. Mr. Carmichael has attempted to arrange these diseases under distinct heads, and thinks he can point out satisfactorily several kinds of venereal sores well characterized, each of which he supposes to arise from a different poison, and to be followed by its peculiar constitutional symptoms.

"He has found all these curable without mercury, except what he calls the syphilitic chancre, and the secondary symptoms which arise from it: viz. the excavated ulcer of the tonsils, the scaly eruption on the skin, and some peculiar affections of the bones. It would certainly be an important improvement in surgery, if such an arrangement could be made, and such a degree of accuracy attained; but the appearances of sores can seldom be relied on, in parts of such vascular structure, and in the midst of sebaceous glands. Peculiarity of constitution must also be taken into account."

"The cachexia syphiloidea, or pseudo-syphilis, to which a great many of these cases are referred by the surgeons of this country, is often a most obstinate and formidable disease. It was not distinguished from syphilis till of late years, and probably some genuine cases of the latter have been treated and cured under such a denomination. Mercury, if carried to any extent, produces in it most pernicious effects; indeed, the cachexia syphiloidea is rarely met with except where that medicine has been freely employed, which is, therefore, considered as one of its exciting causes. It would appear from a remark of Dr. Scott's, however, that that cause is not alone sufficient to give rise to it. He states, 'that during the whole of his residence in India, where mercury is so commonly, so largely, and sometimes so injudiciously, given for affections of the liver, he never knew a single instance of this new disease having arisen where syphilis was certainly out of the question.'

"Like syphilis, the cachexia syphiloidea appears to be produced by some absorbed poison, and frequently follows different sores. I met with a well-marked case of it lately after a very painful and ill-conditioned sore on the finger, and another which followed an ulcer on the lip. It has never been satisfactorily ascertained whether such sores have the power of secreting a matter which can produce the same disease in others.

"There seems, however, no doubt, that in some of its stages the disease itself is contagious. I have known three instances of husbands having communicated it to their wives; and, in two of these, I was not able to ascertain that there had been any sore after marriage, from whence inoculation could have taken place.* The

"* Two cases of this nature are given by Mr. Abernethy, in his Essay on Diseases resembling Syphilis."

same remark, with respect to our ignorance of the means of infection, applies to the disease with which infants are frequently affected shortly after birth, characterized by coppery spots, emaciation, and other symptoms, and supposed to be the effect of the virus of syphilis. We commonly find, in such cases, that one of the parents, or the nurse, have had some venereal or syphiloid disease at no very distant period; but the precise mode of infection can seldom be ascertained.

"The modification which the symptoms of syphilis undergo, from the injudicious use of mercury, so conducted as to fail of effecting a cure, is an inquiry of considerable interest, and would, if properly conducted, throw a great deal of light on the history of the disease."

In our opinion, the impossibility of effecting a cure of the venereal chancre without mercury, remains as fixed as ever. Mr. Pearson has certainly shown, that diseases resembling lues have occasionally arisen. But who, we would ask, suggested any thing *rational* on this subject before Mr. Hunter wrote. It is much to be regretted, that in the army, where so many opportunities occur, the source of these diseases is not better ascertained; we say nothing about modification: that the virus itself, and not the modification, is the cause of the actions excited, appears satisfactorily to us, by Mr. Fergusson's account of the opera dancer, and was remarked more than twenty years ago by another writer in his observations on Mr. Bell. "I should think (says this writer) that Mr. Bell has been treating some morbid poison different from the venereal. What very much strengthens this opinion is a passage *in his account of chancre,* that of seven cases of phagedænic ulcers of the glans penis, he traced four from one, and three from another individual source of infection.*"

---

* To this passage, the following note is annexed, by Mr. Bell:—"This rapid progress, which chancres in some instances make, is, for the most part, supposed to depend upon some peculiarity in the constitution of the patient; for, in general, chancres remain circumscribed, and nearly stationary for a great part of their duration. But I have reason to think, that, in some instances, it proceeds *from the nature of the matter by which they are produced:* I conclude that it is so from chancres of this description, being much more frequent at particular times than at others, and from observing them at the same time in *different people receiving the infection from the same woman.* About two years ago, I met with more instances of this phagedænic chancre in the space of three or four months, than I had seen for several years before, and in four of them the infection was traced to the same woman: the chancres in all of them appeared early, and made such rapid progress, that very

That the cachexia syphilöidea, or pseudo-syphilis, is *often* a most obstinate disease, ought not to be a matter of surprise, till we know which disease, or which form of disease, is meant, by such general, such unmeaning, and consequently such unphilosophical, terms. But we have so much work before us, that at present we must dismiss Mr. Rose, to make room for another army-surgeon, better known by his longer residence among us, and by his former ingenious publications.

The first sentence of his paper is particularly remarkable.

"There are (says Mr. Guthrie) no diseases to which the male sex is so very obnoxious as those of the sexual organs, and there are none which have more occupied the attention of surgeons; yet there is not a subject in surgery of equal importance, on which less has been written since the time of Mr. Hunter. We find that those who have had the greatest opportunities of acquiring knowledge, have, for the most part, refrained from communicating to the public the results of their observations; and that this has arisen rather from the difficulty of the subject than from its being so thoroughly understood as to require no comment, will be immediately acknowledged by every one of discernment and experience."

It is natural to ask, why this cessation of writers on a subject which formerly occupied every pen? Not only, too, have the writers on this disease disappeared, but the works of former writers are gone into disuse. Who now regards Swediaur, once the oracle of Edinburgh?* who now reads Bell? or who quotes either of them on this disease. Of all the numerous opponents of Mr. Hunter, that sprung up on his first publication, which of them is now remembered? What should be the fair inference, but that every future writer would first see whether he has discovered what Mr. Hunter overlooked, or whether his observations are consistent with Mr. Hunter's doctrines; and, if not, how far they are maintainable in opposition to him.

---

troublesome hæmorrhages occurred from them in the space of three or four days from their first appearance; and in a small town to which I was lately called for an alarming hæmorrhagy, produced by an ulcer of this kind, the surgeon in attendance informed me, that in the space of a few weeks he had met with three instances of the same nature, and in which *the infection was also traced to the same woman.*" Here, therefore, the effect of the virus was so similar in so many subjects, and so different from syphilis, as to preclude every suggestion concerning "modification."

* See Cullen's First Lines.

One should conceive nothing more simple than such a mode of proceeding, as we have now only one book to which we need refer, instead of the numbers by which our predecessors were confused. It is thirty-one years since Mr. Hunter published. His doctrines were so perfecly new, that he could hardly expect his equals in age would readily adopt, even if they took the trouble of studying, them. They have had, however, the good effect of relieving the press from the number of ephemeral publications with which it formerly teemed ; and, what is a still happier event, they have relieved many hypochondriacs from the dread they once entertained of the impossibility of ever being free from the complaint,—from the terrors of entailing it on their offspring,—and from repeated salivations, which often ended in insanity.* They have also roused one branch of the profession from that hasty decision, the offspring of indolence and ignorance, by which, at one time, every symptom which could not be easily explained was imputed to this *proteiform*—this *polymorphous* disease, or to some *species* or some *modification* of it. In Mr. Hunter's work, the laws of the venereal disease are defined, and many of its deviations from those laws ascertained. Other diseases, with which it was once confounded, have been separated, and some progress has been made in ascertaining their laws also. Lastly, we no longer salivate with redoubled cruelty, in proportion as we find the disease increase under, or return after, the use of mercury. How much time and paper, then, would be spared, if all this were well attended to, instead of the reiteration of such expressions as *pseudo syphilis* and *cachexia syphilöidea.* We are far from accusing the inventors of these terms as the causes of all the ill-stated facts and unfounded reasoning which have followed. On the contrary, they have done much *molem secare.* But their terms have given authority to expressions which have been abused in other hands. It is true, the writers of the papers before us are of a rank above being led astray by mere words: we can, therefore, only regret that they have not studied Mr. Hunter with greater attention.

Mr. Guthrie observes, that the only means by which he can elucidate a subject beset with so many difficulties, would be to adopt the opinion of a French author, who asserted that there is not, nor ever was, such a disease as the venereal. This is bold, when so many able writers have witnessed the progress of local complaints with an uniform character, which yielded only to mercury, and have never

* See Haslam on Insanity.

resisted that remedy. On the continent, we are told, little attention is paid to the primary appearance of sores. Was not such the case with too many in England before Mr. Hunter wrote, and for some time after. Since his time, "surgeons," we are told, "prided themselves on their peculiar talent in distinguishing those ulcers which absolutely required the use of mercury for their cure from those that did not; but the value of this prescience will be more duly estimated, now that it has been ascertained that every sore, of whatever description it may be, will heal without its use, provided sufficient time be granted, the constitution be good, the patient regular in his mode of living, and that attention be paid to cleanliness and simple dressing, and to keep the patient in a state of quietude."

During the last eighteen months, all the ulcers on the penis at York Hospital have been treated without mercury, and all have been healed, most of them having skinned over rapidly. The period when their amendment commenced has been various. A great question remains, whether sores so healed were more liable to secondary symptoms? According to the opinions commonly entertained, we are told there ought not to be a doubt on the subject. We pretend not to know what the common opinion may be, but certainly such would not be Mr. Hunter's opinion. On the contrary, there was a greater probability that such sores were not only not venereal, but that they were not even the effect of any morbid poison whatever; and, consequently, that no secondary symptoms would follow. If by rapid skinning is meant the healing of the part without previous granulations, this may be admitted among the characters of a morbid poison, but is not peculiar to the venereal. Has it not also been long since conjectured, that some morbid poisons never shew secondary symptoms, excepting when the primary have been interrupted by mercury?

The following passage we select in justice to Mr. Guthrie, as it shows that he has Mr. Hunter's doctrines in view, though he sometimes thinks it unnecessary to advert to them:—

"In regard to secondary symptoms, it appears that they occur after primary ulcers which have not been cured by mercury; and that they do also occur after a well-regulated course of mercury, there is no man of experience will, I believe, deny. Indeed, Mr. Hunter, whose accuracy in matters of fact will not be disputed, has left us, through his commentator Dr. Adams, a most interesting case of this nature, where the disease not only affected the first, but also the second, order of parts, although mercury was each time properly exhibited for its cure. This case is given as explanatory of Mr. Hunter's doctrine, that, if the disposition for the disease be formed, mercury cannot cure it until it come into action; which, in plain language, means nothing more than that the disease cannot be prevented, in certain constitutions, from running its own course, when it may at last be cured."

We are glad of any language which illustrates Mr. Hunter's doctrines, and the above is a very just, though to us a new mode of expressing them.

We reluctantly pass over several other remarks, to find room for the author's concluding paragraphs:—

"1. Every kind of ulcer of the genitals, of whatever form or appearance, is curable without mercury. This I consider to be established as a fact, from the observation of more than 500 cases which I am acquainted with, exclusive of those treated in the different regiments of Guards, and which occurred in consequence of promiscuous intercourse.

"2. Secondary symptoms, (and I exclude trifling pains, eruptions, or sore throats, that have disappeared in a few days,) have seldom followed the cure of these ulcers without mercury, and they have, upon the whole, more frequently followed the raised ulcer of the prepuce than the true characteristic chancre of syphilis affecting the glans penis.

"3. The secondary symptoms, in the cases alluded to, amounting to one-tenth of the whole, and which were treated on the antiphlogistic plan, have hitherto been nearly confined to the first order of parts; that is, the bones have in two cases only been attacked, and they have equally been cured without mercury.

"4. As great a length of time has elapsed in many of these cases without the occurrence of secondary symptoms, as is considered satisfactory where mercury has been used,—viz. from six to eighteen months.

"5. The primary sores were of every description, from the superficial ulcer of the prepuce and glans to the raised ulcer of the prepuce, the excavated ulcer of the glans, and the irritable and sloughing ulcer of these parts. In the inflammatory stage attended by itching, scabbing, and ulceration, they were treated for the most part by antiphlogistic and mild remedies; in the latter stage, when the ulcers were indolent, whether raised or excavated, by gentle stimulants.

"6. The duration of these stages is very different, is often increased by caustic and irritating applications, and is much influenced by surgical discrimination in the local treatment.

"7. The last, or indolent stage, often continues for a great length of time, especially in the excavated chancre and raised ulcer of the prepuce; and it appears to me that, in these particular cases, a gentle course of mercury, so as slightly to affect the gums, will materially shorten the duration of it, although in others it is occasionally of no service.

"8. Although the secondary symptoms do for the most part yield to simple remedies, such as venæsection, sudorifics, the warm-bath, sarsaparilla, &c. without much loss of time,—that is, in the course of one to four and six months; yet, as in the primary ulcers, a gentle course of mercury will frequently expedite, and in particular persons and states of constitutions is necessary to effect, a cure; and that a repetition of it will even, in some cases, be requisite to render it permanent."

These facts are stated with great candour, and of their truth, as far as the author's late observation extends, there can be no question. We shall defer any other remarks till we have noticed an article in the

*Edinburgh Medical and Surgical Journal*, No. LIII. January, 1818.

*Observations on the Treatment of Syphilis without Mercury.* By John Thomson, M.D. Professor of Surgery to the Royal College of Surgeons at Edinburgh, &c. and Surgeon to the Forces.

Dr. Thomson informs us, that, by a careful investigation into the history of syphilis, he was some years ago led to doubt whether mercury, in some of its forms, is the only safe, effectual, and specific remedy for the cure of that disease.

"These doubts were much increased by the discussions to which the various communications made to the late Dr. Beddoes gave rise, respecting the efficacy of nitric acid in venereal complaints; by the appearance of Mr. Abernethy's valuable publication on the diseases resembling syphilis; and by conversations, at different times, with my friend Mr. Pearson of the Lock Hospital, as well as by the perusal of notes taken from his excellent lectures upon that subject. In the uncertainty in which I was respecting the proper diagnostic marks of constitutional syphilis, I resolved, in the treatment of those cases that should come under my care, in which mercury had had a full trial, and particularly in which it seemed to have produced injurious effects, to abstain altogether from prescribing that remedy, till a trial should be made of some of the other remedies which had at different times acquired reputation for the cure of venereal complaints. That which I made choice of was the simple decoction of sarsaparilla; and, after a very ample employment of this substance, I feel myself compelled to adopt the opinions of some of the earlier writers on the venereal disease, with regard to the singular efficacy of this root in curing symptoms which have usually been reputed syphilitic; and also, with a few exceptions, to believe in the justness of the conclusions to which the late Sir William Fordyce had been led from an extensive trial of sarsaparilla. I have employed this remedy in every form of the disease, which either remains after, or succeeds to, the use of mercury, and have had the satisfaction to observe all manner of cutaneous eruptions and ulcerations; ulcerations of the throat, pains and swellings of the joints and ligaments, and nodes of the bones, gradually disappear under its mild operation, when its use was duly persisted in, and was at the same time accompanied by attention to regimen, and the proper local treatment. In particular cases, the recovery has been tedious, and it has been necessary to have recourse to the use of sarsaparilla a second or even a third time. I may, however, remark, that I never had occasion to see the venereal diseases in which it was employed make those rapid and alarming advances which we see so often occur in them during the use of mercury; nor am I aware of any permanently injurious effects which the sarsaparilla produces, either immediately or remotely, upon the constitution."

We cannot help expressing our surprise that Dr. Thomson should not have learned from Mr. Hunter how many diseases on the genitals, as well as secondary symptoms, are curable without mercury. This is all that can be inferred from either Mr. Pearson or Mr. Abernethy, from both whose writings the reader is left to conclude that the true venereal ulcer yields to that remedy only. Dr. Beddoes' cases are given in so desultory a manner, that no inference can be drawn from them: of their fallacy it is enough to remark, that the acids as a cure soon fell into disuse. The general result of Dr. Thomson's paper is, that sarsaparilla is the best remedy for all those local diseases, primary or secondary, which are considered venereal. The authority of Sir William Fordyce will be found meagre enough by those who have patience to peruse him, as the greater part of his cases were such as had resisted mercury. Dr. Thomson conceives himself fortunate in meeting with a Tract, published in 1709: unless it contains more information than he produces, we should conceive the only value of the work is in its antiquity and the obscurity of the author.

That the venereal disease is much more rare than has been generally suspected, has long since been remarked by all the disciples of Mr. Hunter. That gentleman, during his life-time, was said never to admit that any one was poxed. This was only a figurative mode of expressing his scepticism on that subject; but it shows how large a proportion of suspicious productions he weeded from this prolific garden. It now seems remarkable how little effect he produced on the general practice, notwithstanding the great celebrity of his name. In this, however, he is not singular. The cold treatment of small-pox was never generally adopted by the contemporaries of Sydenham, nor by his successors, till the boldness and success of the Suttons *forced* it into general notice; and then, with a blind enthusiasm, equalled only by the former scepticism, cold was conceived a specific against this formidable disease.

Having devoted a considerable part of this Number to a subject which at present so much interests the public attention, our readers will not be dissatisfied by a short historical reference, for which, we trust, their mind is in some measure prepared.

Whoever carefully peruses the ancient writers, sacred or profane, will find a certain degree of obloquy always attached to cutaneous diseases of every description, but most of all to diseases about those parts which, for the sake of decency or modesty, are kept concealed. Cutaneous diseases are often the effect of vermin, or attendant on extreme poverty; and certain parts are disposed to fall into disease from mere inattention to cleanliness. Hence, probably, the disgrace attached to each. Even among the Jews, emrods in the secret or in the hinder parts was a great reproach. In the Latin satirical poets, we meet with references to cutaneous diseases, which rendered the sufferers ridiculous or odious; yet only the coarsest among them refer to complaints about those parts which were concealed. Juvenal and Perseus both mention *fici*, sometimes with ridicule, sometimes with disgust; but we have no mention of *obscæ narum partium vitia*, excepting by Celsus, who, though a medical writer, apologises for introducing them, remarking, that the Greek terms were more delicate, not being so vernacular. Cicero seems to have availed himself of them, when referring to the Epicurean philosophy. "*Dicat* δυσεριϰα ϰαι δυσεντεριϰα παθη *sibi molesta esse, quorum alterum edacitatis, alterum turpioris intemperantiæ, &c.*—"*

It is not for us to assert that the ancient physicians had not discriminated cutaneous diseases with so much accuracy as to apply the numerous words lately adopted from them to some specific and well-ascertained meaning. If they *had*, however, we are not ashamed, in such company as the late Heberden,—a scholar, a physician, and an honest man,—of acknowledging our incapacity to unravel their meaning.† The same may be said of the leprosy of the middle ages. Yet we affect to affix certain marks to each, whilst we can rarely find terms in our own language satisfactorily to con-

* Epistol. It is true that all the authors about the Augustin age were fond of interlarding their more familiar writings with Greek, but it is at least fair to consider the above as an illustration of Celsus' remark on that subject.

† See his chapter *Cutis vitia.*

vey a description of complaints which are perpetually before our eyes. Till the fourteenth century, we have seen that the common appellation for cutaneous diseases was leprosy; and, to this day, persons are often said to look like lepers, by those who, if asked their meaning, would be unable to explain themselves. The venereal disease has, however, for a succession of years, been the common resource where no other name could be conveniently found. Astruc did something to correct this negligence, but afterwards, by introducing *Pandora's box* as an illustration, he left the question as unsettled as before. In these days, it may surprise us that such a writer as Lancisi, after speaking of aneurism as sometimes hereditary, should derive it in such cases from syphilis. But Pandora and Proteus gave a fair *licencia poetica* to those authors who did not profess to elucidate this disease.

We pass over all the other writers from Astruc, to introduce Mr. Hunter; for, excepting some valuable practical remarks by Howard, we know of none between Astruc and Hunter that are worthy of notice. Such, indeed, by the remark extracted from Mr. Guthrie, seems to be the general opinion.

Mr. Hunter was one of those few men who are born to improve natural science. A mind like his would at once see that the whole difficulty consisted in the want of a proper mode of conducting the enquiry; and that he had only begun his work when he ascertained the true character of that disease which would yield only to mercury, and which, according to its inveteracy,* required a proportionate irritation from that remedy. When this was satisfactorily determined, it was no longer impossible to detect its occasional aberrations and their causes, nor to distinguish them from other diseases on the same parts, or on other parts (the mouth and the nipples), arising from an intercourse between two subjects. He found that some would heal with only common remedies,—others with a very slight exhibition of mercury; whilst the true chancre yielded to nothing but mercury, and, if at all inveterate, required such an excitement from that remedy as would exasperate most, if not all other, local diseases.

On this occasion, it may be right to draw the attention of our readers to the term *chancre*. This is well known to be

* I hope the reader will pardon me if I mention, that, by inveteracy, nothing more should be inferred than the *length of time* that the symptoms have existed.

only another word with the French for cancer.* Now the French, (to the time of Astruc, at least,) retained the true classical meaning of the word cancer,—that is, they applied it to any local disease which would not yield to the powers of the constitution, assisted by common remedies.† This being the case, it is evident that, before and at the time of Astruc, such a disease existed, and could be cured only by mercury. That it has continued to our own times can hardly be questioned, when we consider the general scepticism of Mr. Hunter concerning so many complaints during his day confounded with the venereal, for which he deprecated the use of mercury; and when we add to this the attempts at the Lock and other hospitals, to cure chancres by opium, by guaiacum, by sarsaparilla, and by chlorine, —their failure in all, and their successful return to mercury; to these we may add the temporary substitution of Vanswieten's corrosive sublimate, Chapman's calomel, and many others who have conceived slight doses of mercurial salts sufficient, because they found them so in most of the ulcers on those parts. Yet, all this while, it is easy to see that a disease existed of a determined character, which would yield only to a violent mercurial irritation; and the most experienced writers agree in the character of that disease, if they undertake to describe it at all.

We are ready to admit, not merely for the sake of argument, but to offer it as our conviction, that the true venereal primary ulcer, or chancre, is more rare than it was,

* This manner of softening the *c*, followed by the vowel *a*, especially at the beginning of a word, is very common in that language. We cannot assert that it is not universal. The following words occur to us:—chamber, from *camera*; chemin, from *camena*; chemise, from *camisa*; and several other words, noticed by Mr. H. Tooke in his remarks on the French adverb *chez*.

† Omnis autem cancer non solum id corrumpit, quod occupavit sed etiam serpit; deinde aliis aliisque signis discernitur.— Cels. lib. iii. cap. 26. Almaloveen has a note, which it may be right to cite as a proof that learning and diligence are not sufficient for interpreting, however they may serve for collating or comparing authors. He conceives, that Celsus refers only to the difficulty of distinguishing cancer in its occult stage, and, confounding modern theories with the language of the ancients, cites Galen's remarks on Scirrhus as a proof. But, whoever divests himself of modern technicalities, will perceive that Celsus meant no more than to show the richness and accuracy of the Greek language, in possessing terms appropriated to the various species of cancer, the genus of which he accurately defines.

even in Mr. Hunter's day; nor is it difficult to see how this should happen. The improved state of society renders it easier for every class of both sexes to gain relief: there are, consequently, fewer females under this disease consigned to harlotism. How then, it will be asked, should these other morbid poisons, if they are such, be more common? We know not that they are more common; but it will be enough that they still maintain their frequency, and are no longer mistaken for venereal. If, indeed, we view many of them only in the male organs, we should conceive it impossible they should ever spread at all, because such is the condition of the part that coition must be painful, if not impossible; whereas, with a recent chancre, it may be performed without difficulty. But we have not yet ascertained the state of the female organs, in any but the true venereal chancre. We have seen that the opera-dancer, whilst she was making such havoc among her lovers, was not only enabled to continue her amours, but even her occupation on the stage. Among the tooth and nipple cases related by Mr. Hunter, there is no reason to believe that any of the first, nor many of the latter, subjects, from whom the poison was received, were diseased themselves. All these difficulties were nothing whilst *Pandora* and *Proteus* were admitted into the *materia morbida*. But Mr. Hunter has obliged us to look closer into the question, and we have no reason to doubt that the present enquiry will accomplish what was well known to him,—what he taught with accuracy,—but what, like many other doctrines, is only neglected, misunderstood, or misinterpreted, on account of its simplicity; that is, because they oblige us to attend minutely to every phenomenon, its series, and order.

That, in the time of Celsus, these diseases were frequent, both in Rome and Greece, is evidently implied in the passage we have selected; but we have no account of their existence in the *vagina*, nor does that accurate writer impute them to coition. For this reason, it is less remarkable that, though he describes secondary symptoms in the skin and in the bones, yet he no way connects them with the *colis morbi*, nor seems to have had any conception that they arose from such a cause. This chain of symptoms, however, by the case of John of Malmsbury, does not seem to have been entirely overlooked before the venereal disease arose.*

From all that has been said, it appears—that cutaneous

* See Mr. Becket's paper in the Philosophical Transactions, and also Astruc's Abridgment.

diseases have been more general in proportion as the inferior classes of mankind have been more oppressed by their government, or by privations from unfavourable seasons, against which no provision has been made; that these diseases have, by common historians, been included under general terms, though some of them have been discriminated by more attentive medical writers; that less attention was paid to them before the introduction of Christianity, excepting among the Jews, because the inferior ranks were mostly slaves, and considered as unworthy of notice; that, during the corrupt state of Christianity, the priesthood, availing themselves of every means of acquiring wealth, added leprosy to their other sources. As that disease had never been accurately defined,—as the word had been, by the Arabians, applied to the elephantiasis of Aretæus, and to most cutaneous complaints,—and as elephantiasis has been, by the moderns, applied to a swelled leg and foot,—some nosologists have most unaccountably puzzled themselves under which class they shall designate diseases they have never seen, and which have never been accurately defined, and, by introducing still more terms of uncertain import, have added greatly to the confusion. That it is probable cutaneous diseases have always occurred as the secondary symptoms of ulcers on the genitals, but that they were not suspected to arise from such a cause, and that they were even much less frequent when the primary symptoms were treated without mercury. That the introduction of the venereal disease, in which secondary symptoms were accurately traced, induced a general opinion that all other diseases, following a similar progress, though differing in character, were the same; and hence the same remedy was applied, sometimes with immediate success, with slower effects on others, and that it exasperated a few, and proved the cause of secondary symptoms: but that, during the whole of this latter period, the most accurate writers distinguished the true venereal character, and found it would yield only to mercurial irritations, excited higher in proportion to the length of time which the ulcer had existed. That Mr. Hunter was the first to discover that secondary symptoms on the skin, though they could not be prevented by mercury, might be more easily cured than the primary chancre; and that new morbid poisons are perpetually arising, which resemble the venereal in the manner in which they induce primary and secondary symptoms, but that they all differ considerably in the character of the first, and some of them of the second also. That, from the nature of these diseases, and the improved state of society, it is probable the venereal will be gradually exterminated; but that the other poisons will continue, till we are better acquainted with their causes, and even increase as those parts become more generally free from the venereal virus.

Such appears to us the present state of the question, in which we cannot but congratulate the rising generation on the probability of a more general attention to, and comprehension of, Mr. Hunter's doctrines: that not only his doctrines will be understood, but the advice will be properly attended to, with which he concludes his invaluable practical work, and with which we shall close this long article:—

"I cannot," says he, "conclude without intimating that undescribed diseases, resembling the venereal, are very numerous; and that what I have said is rather to be considered as hints for others to prosecute this enquiry further, than as a complete account of the subject."—*Hunter on the Venereal Disease, last paragraph.*

---

*The Influence of the Atmosphere, more especially the Atmosphere of the British Isles, on the Health and Functions of the Human Frame; embracing Observations on the Nature, Treatment, and Prevention of the principal Diseases resulting from sudden Atmospherical Transitions; and unfolding Original Views and Fundamental Principles for the Prolongation of Life, and Conservation of Health. To which are added, Practical Researches on the Pathology, Treatment, and Prevention of Gout and Rheumatism in all their Proteian Forms. An Essay.* By JAMES JOHNSON, M.D. Surgeon to his Royal Highness the Duke of Clarence; Author of the "Influence of Tropical Climates on European Constitutions;" and one of the Editors of the Medico-Chirurgical Journal and Review. London, 1818. 8vo. pp. 290.

THE writer of the following volume already possesses a strong claim upon the approbation and esteem of the professional public, by his late work on the Influence of Tropical Climates.

In the volume before us, the author first divides the principal sympathies that obtain in the human body, into four sections. Those are prefaced by some introductory remarks upon the general constitution and influence of the atmosphere, in which he observes, (page 10) that "it is only in tropical regions where hepatic diseases force themselves upon the most inert observation, that the sympathetic connection between the functions of the skin and liver would be likely to arrest attention, particularly where the personal sufferings of the observer might be the means of exciting a more than usual degree of accuracy in the investigation. Such was the case when I traced the sympathy in question; and by which I have been enabled to delineate the nature, the causes, and the treatment of diseases in hot climates." An observation, the accuracy of which we have ourselves confirmed, when under circumstances precisely similar to those stated by the author.

Having drawn the attention of the reader to some original and highly judicious observations on the best means for preserving the constitution from the effects of climate, our author proceeds to the consideration of those numerous affections of the lungs derived more or less remotely from atmospheric vicissitude. These affections are distinguished as resulting from the *cutaneo-pulmonic* sympathy. His mode of treatment in these complaints, is marked with that decision, the importance of which is not sufficiently appreciated in the management of acute disorders.

The *cutaneo-gastric* sympathy is comprehended in the second section, in which the author states his having seen three distinct cases of gastritis from the sudden application of cold to the surface of the body, which observation is followed by some strong illustrations and ingenious remarks upon the connection that may be frequently observed between eruptive complaints and affections of the stomach.

The third section includes the consequences of the *cutaneo-intestinal* sympathy, under which head the author first considers inflammation of the intestines, and introduces some most excellent practical remarks. In drawing the comparison between inflammation of the external, and that of the internal coat of the intestines, he has furnished us (in page 31) with a most clear diagnosis.

The mode of treatment he recommends in these complaints, is evidently the result of his own personal very accurate observation.

In the consideration of the various circumstances that relate to the treatment of dysentery, many original conclusions occur, which can only be perfectly understood by reference to the work itself.

We suspect many of our readers will object to Dr. J.'s opinion concerning the cause of cholera morbus, that the complaint does not depend on increased secretion of bile; although the ground the author has taken up on a very extended field of observation, entitles him to due consideration.

In the fourth section, in which the more striking consequences of the *cutaneo-hepatic* sympathy are noticed, our author enters first upon the consideration of Hepatitis, of the symptoms of which he traces all the occasional irregularities with so masterly a hand, that, though his treatment be regulated, it is by no means common, and is entitled to a serious attention.

The consideration of chronic hepatitis next follows, on which, after many pertinent remarks on the probable office, as well as principal sympathies of the liver, the author passes on to an enquiry into the causes and best mode of treatment for the various morbid affections to which the biliary system is subject; succeeded by some observations upon *cardi-hepatic* complaints, with the effect of atmospheric influence in laying the foundation for scrofula, and in producing fever. Our author is not disposed to admit the opinion lately brought forward, that every case of fever is connected with local inflammation—a sweeping position, which was never likely to gain credit with the profession at large.

The remaining pages of this part of the work, relating to the proximate cause, the consequence, and the treatment of fever, are replete with practical remark and criticism upon some of the opinions of the present day.

The second part of the work relates to the preservation of health, or the prevention of disease, and is conducted in a most interesting and satisfactory manner. The heads under which it is arranged are,—food, drink, exercise, clothing, ablutions, cold and warm bath, the passions, *morbi cruditorum*, sleep, and lastly medicine.

The remaining portion of the work is introduced under the form of appendix; the first part of which contains practical researches upon gout, in all its various forms, translated and condensed from the French of M. M. Guilbert and Hallé, as drawn up for the *Dictionnaire des Sciences Medicales*, in 1817.

The variable mode of attack gives occasion for five divisions. First, regular or acute articular gout; second, chronic or irregular gout; third, primitive asthenic gout; fourth, fixed gout; fifth, primitive fixed gout. For our parts we are satisfied with the division of chronic and acute. The practical remarks are however useful.

Among the curious cases that are detailed, we shall take the liberty to extract the following, as proposing to our consideration a point hitherto unexplained, and in general, we may add, unadmitted.

"*Case.*—A lady, arrived at the turn of life, became affected with *periodical* hypochondriasis; each fit of which commenced at sunrise, increased till noon, when it was at its acmé, and then declined as the sun approached the western horizon. The degree of hypochondriacal affection bore a relative proportion to the altitude of the sun.—The patient, at first merely a little low-spirited, became gradually more and more dejected and oppressed, till she appeared devoured with chagrin, or overwhelmed with despair. But, when the sun had attained the zenith, such a sentiment of terror took possession of her mind, that the least noise,—the lowest spoken word, seemed to her imagination the signal of some dreadful misfortune that was ready to burst over her, and destroy her! As the sun declined from the meridian, this morbid affection diminished; retracing its steps through the same grades of sadness which marked its development. To the state of terror succeeded a profound sense of chagrin, which, becoming weaker and weaker, took on various shades of sorrow, till it ended in a mild and not unpleasant state of melancholy. Such was the train of mental emotions; during which, other phenomena presented themselves. At the moment of accession, the patient felt a sensation of darting or quivering *(tiraillement)* in the region of the heart; which sensation radiated in all directions, but particularly towards the superior parts. Then the pulsations of the heart, the cœliac, and finally all the other arteries, became hard, strong, and greatly accelerated. Such was the augmented propulsion of blood into the arteries, that we could see the fingers start involuntarily at each systole of the heart. This curious sensation *(tiraillement)* in the cardiac region, and which *preceded* the preternatural action of the heart and arteries, increased *pari passu* with that action, with the hypochondriacal paroxysm, and with the elevation of the sun above the horizon."

The above case we have selected, not because it is more practically useful than very many others to be found in this work, but on account of its connexion with the author's opinions concerning the movements of the spheres. An opinion which ought not to be hastily discarded. For the conclusions derived from the above case, we must refer to the work itself.

The illustrations (by cases) brought forward of each kind of gout are numerous and valuable, affording ground for the division that has been adopted; the varieties of symptoms that the disease may assume are said to be almost literally so infinite, that it is only by very attentive application that the reader can make himself master of the whole.

Upon the morbid anatomy of this terrible disease, several most interesting and minute dissections are related, and the general conclusion drawn from them is, that the effects of articular gout are not confined to this or that tissue, but that they may affect them all, either separately or collectively.

The nature of the earthy deposit in gouty subjects is shown, by chemical analysis, to be various; it is found to be urate of soda, urate of lime, phosphate of lime, carbonate of lime, either singly or in union with each other, and a certain proportion of animal matter.

Some interesting remarks are also made upon the presence of disengaged acid in the urine and perspiration preceding the attack of gout; after which, follows the enumeration of causes, and the prognosis; the latter, of course, variable, according to the circumstances of constitution and disease.

As to the *nature* of gout, it is supposed to be dependant upon a morbid affection of the lymphatic system; and the principal argument brought forward in support of this conclusion seems to be, the universal diffusion of the lymphatic vessels, which alone is supposed adequate to explain the variety and extent of the disease; but it appears to us, that this reasoning is at least unsatisfactory, for it is very plain that no system can be more extensive than the arterial, and it is still doubtful with some, if the lymphatic system is so universally extended as the vascular—a doubt which our present knowledge of anatomy will not unravel.

Under the head of Treatment, a very interesting discussion is entered upon with regard to the value of the different remedies that have, in various ways, been useful in preventing or relieving the attack. The empirical treatment—Pradier's cataplasm, Paulmier's remedy, eau medicinale, the remedy of Hald, methodic or regular treatment, cold, are all considered, and form an interesting epitome of every thing that is necessary to hold in view at the bedside of the arthritic patient.

The consideration of the Hygëia Arthritica forms the last part of this valuable treatise, every page of which is so concentrated, that the translator deserves infinite credit for that application and industry by which he has brought into the space of eighty-one pages the whole interest of a much more extended octavo volume, thereby facilitating the acquisition of useful knowledge on a disease so complicated, and so difficult to be understood.

Some practical researches on the pathology, treatment, and prevention, of Rheumatism, form a second appendix to the volume. In these, the author has brought several important points forward relating to the history of rheumatism, which, although previously not unknown, were too little considered. In this Essay, Dr. Johnson has not only given the results of his own experience, but, by the assistance of his friends, has been enabled to add those of several gentlemen whose professional talents are well known, and whose united exertions have certainly given a peculiar value to the observations.

The principal feature in this Essay, is the remarks on translation of rheumatic action to internal parts, and particularly to the heart; insulated histories of which are to be found thinly scattered in late publications; but, in the present Essay, the reader is enabled to see and reflect upon the whole of the information hitherto acquired, condensed into a single focal point.

Such is our imperfect account of a work, which does honour to the talents and industry of the author, and which certainly contains more useful information than any other to be procured on such easy terms.

---

